# Unpacking the blood flow restriction device features literature - set/interface pressure: recommendations and considerations for an evolving field

**DOI:** 10.3389/fphys.2025.1627583

**Published:** 2025-06-18

**Authors:** Nicholas Rolnick, Sten Stray-Gundersen, Victor S. de Queiros

**Affiliations:** ^1^ Department of Exercise Science and Recreation, CUNY Lehman College, New York, NY, United States; ^2^ The Human Performance Mechanic, New York, NY, United States; ^3^ The BFR PROS, New York, NY, United States; ^4^ Department of Exercise Science, Arnold School of Public Health, University of South Carolina, Columbia, SC, United States; ^5^ Department of Physical Education, State University of Paraíba, Campina Grande, Paraíba, Brazil

**Keywords:** Delfi, autoregulation, resistance training, limb occlusion pressure, arterial occlusion pressure

## 1 Introduction

Blood flow restriction (BFR) training—defined as the use of external pressure to partially restrict arterial inflow and intermittently occlude venous return during low-load exercise—continues to expand in both clinical and athletic settings (Scott et al., 2023). While continuing to evolve, there is increased attention being paid to how the BFR stimulus itself is impacted with different device features. Early literature focused on device features such as cuff width (Laurentino et al., 2016) and cuff material (Buckner et al., 2017) and their impact on acute responses and longitudinal adaptations to BFR exercise. Newer focus has shifted to autoregulation of applied BFR training pressures (Jacobs et al., 2023; Rolnick et al., 2024a; Rolnick et al., 2024c), bladder design (Rolnick et al., 2025; Rolnick et al., 2024b), and cuff shape (Vehrs et al., 2024).

## 2 Clarifying the terms and the case for interface pressure control

However, one feature that is often not recognized but is receiving a growing amount of attention is the set/interface pressure capabilities of a BFR cuff (Hughes et al., 2018; 2024; Neal et al., 2023). This BFR device feature focuses on the stability through which pressure is delivered to the limb and monitored during BFR exercise. The “set pressure” is the pressure that is set by the user whereas the “interface pressure” is the actual pressure exerted by the cuff on the surface of the limb (Rolnick et al., 2023). For example, the set pressure prescribed during BFR exercise could be measured at 200 mmHg which could equate to 80% of the *intended* limb occlusion pressure (LOP) – the minimum amount of pressure to fully occlude blood flow to a limb and is the typically recommended way single-chambered non-elastic BFR cuffs apply pressure in BFR exercise (Jessee et al., 2016; Patterson et al., 2019). All commercially available BFR devices report the applied pressure to the limb in the units of mmHg, making these units the most accessible to providers and researchers to measure the set pressure. On the other hand, interface pressure has not been reported outside of methodological studies (Hughes et al., 2018; 2024) as it requires specialized equipment such as pressure sensors embedded within the BFR cuff or fastened to the underlying limb (Hughes et al., 2018; 2024; Neal et al., 2023). Therefore, counter to set pressure, those practicing BFR do not have access to interface pressure data other than what can be inferred by one of three existing studies (Hughes et al., 2018; 2024; Neal et al., 2023).

It could be reasonably hypothesized that the interface pressure, rather than set pressure, more closely relates to the physiological stimulus imposed by BFR as it is more representative of the force exerted upon the limb during exercise, whereas set pressure is a resting value that is dependent on material, width, ability to autoregulate, bladder design, etc. (Hughes et al., 2024). A non-elastic BFR cuff capable of maintaining a consistent set/interface pressure could be advantageous in research settings to decrease intra- and inter-participant variability to a BFR intervention, mitigate vascular or nervous system stress, and possibly, enhance longitudinal responses.

To date, three publications have implemented some form of set/interface pressure monitoring into their methodology (Hughes et al., 2018; 2024; Neal et al., 2023) (Table 1). Two of them investigated the impact of different BFR cuffs on both physiological and perceptual (Hughes et al., 2018) and maintenance of set/interface pressure prior to, during, and post-exercise (Hughes et al., 2018; 2024). The third employed a strap-based BFR cuff and sought to determine the relative magnitude of pressure applied to the limb under different strap settings (Neal et al., 2023). Evidence from Hughes et al. (2018), Hughes et al. (2024) indicates that only one BFR device–the Delfi Personalized Tourniquet System (PTS) – has the capability to regulate the set pressure effectively to allow for a consistent interface pressure at rest, during exercise, and immediately post-exercise 100% of the time (Hughes et al., 2018; 2024). Other cuffs have shown large variations in their capacity to maintain a consistent pressure on the limb throughout exercise and have exhibited pressure drifts between the set/interface settings (Hughes et al., 2018; 2024). In a recent study, only one other cuff–Suji BFR–was able to maintain mean pressure on the limb post-exercise, albeit with higher variability than the Delfi PTS (Hughes et al., 2024). Other commercially available cuffs including SmartCuffs, B Strong, and Saga Fitness were under pressurized post-exercise and were only able to maintain set/interface pressure consistently 43%–68% of the exercise duration (Hughes et al., 2024). Neal et al. (2023) reported on the average applied pressure using the Hytro BFR wearable clothing with mostly mild variability (<15 mmHg) in applied pressure in all five strap settings. However, the absence of understanding the set pressure due to the elastic, practical-based nature of the BFR cuff limits its applicability in the current discussion. Collectively, these findings suggest that interface pressure regulation *may* play a critical role in the consistency, safety, and physiological effectiveness of BFR application and warrants greater consideration in both research design and practical implementation.

**TABLE 1:** Characteristics of the studies.

Study	BFR cuffs used (width)	Interface pressure assessment	Protocol details	Main outcome measures	Key findings
Hughes et al. (2018)	RI (Hokanson): 13 cm, PT (Delfi PTS): 11.5 cm, HS (Occlusion Cuff): 8 cm	Universal interface device with Pasco Capstone software and quad pressure sensors with participants in supine	*Resting Trial*: 40% and 80% LOP	Interface pressure deviation (rest and exercise), pain (0–10), RPE (6–20), mean arterial pressure (MAP)	*At rest*:
					- RI: −5 mmHg (40% and 80% LOP)
					- PT: −8 to −9 mmHg
					- HS: −20 mmHg (40%), −37 mmHg (80%)
					*During exercise*:
					- RI: +10 to +11 mmHg
					- PT: No difference
					- HS: +37 to +62 mmHg
			*Exercise Trial*:		*Pain*:
			*Exercise/Load*: 4 sets of unilateral leg press at 30% 1RM		- RI and HS > PT (sets 3 and 4, p < 0.01)
			*Repetitions/Rest*: 30/15/15/15 reps with 30 s inter-set rest		- Max pain: HS = 8.3, RI = 7.9, PT = 5.7
			*LOP*: 80%		*RPE*:
			*Cadence*: 2 s concentric, 2 s eccentric		- RI and HS > PT (set 4: RI/HS = 17, PT = 15)
					*MAP*:
					- RI and HS > PT at 1-min and 5-min post-exercise (+10–11 mmHg)
Hughes et al. (2024)	Delfi PTS (11.5 cm), B Strong (7 cm), SmartTools (10.14 cm), Saga (10.15 cm), Suji (10 cm)	Cuff pressure recorded continuously via pressure transducer and LabVIEW with participants in supine	*Duration/Frequency*: 5 BFR systems tested in randomized crossover across 3 visits	Autoregulation (% of time within ±15 mmHg), pressure deviation at end of exercise	*Autoregulation (% total BFR time)*:
			*Exercise/Load*: Single-leg horizontal leg press using Weider Ultimate Body Work Machine with 3 resistance bands		- Delfi PTS: 100% ± 0%
					- Suji: 63% ± 24%
			*Repetitions/Rest*: 30/15/15/15 reps with 30 s inter-set rest		- B Strong: 48% ± 36%
					- SmartTools: 47% ± 31%
			*LOP*: 80% or 300 mmHg (B Strong)		- Saga: 43% ± 31%
					*End-of-exercise cuff pressure deviation*:
			*Cadence*: 2 s concentric, 2 s eccentric		- Delfi PTS and Suji: maintained target but Suji had ↑ variability
					- B Strong, SmartTools, Saga: significantly lower than target (p < 0.05)
Neal et al. (2023)	Garment-integrated BFR (Hytro), dual 5 cm wide elastane straps	Air bladder with Kikuhime transducer placed under strap (strap levels 1–5); mean pressure measured for each level in dominant leg while participant was in standing	*Duration/Frequency*: 6-week home-based program with 2 sessions/week	Mean pressure exerted by strap; knee isokinetic peak torque; DEXA-based lean/fat mass	Thigh girth: 57.2 ± 4.3 cm
					Max strap pressure (level 5): 178.2 ± 12.5 mmHg
			*Exercise/Load*: 3 exercises (bodyweight squats, glute bridges, calf raises)		Max strap pressure (level 4): 158.0 ± 14.0 mmHg
					Max strap pressure (level 3): 118.9 ± 11.2 mmHg
			*Repetitions/Rest*: 4 sets per exercise (30/15/15/15 reps), 30 s inter-set rest, 2 min rest between exercises		Max strap pressure (level 2): 81.9 ± 14.4 mmHg
					Max strap pressure (level 1): 60.4 ± 9.6 mmHg
			*BFR Protocol*: Straps (5 cm) tightened to 7/10 perceived pressure and remained strapped during each 4-set block and unstrapped between exercises		Adherence: 98.5%
					Side effects: 1 excessive pain during (0.7%), 2 bruising (1.5%), 1 paresthesia (0.7%)
					Knee extension peak torque: +12.4 Nm; normalized: +0.1 Nm/kg
					No meaningful changes in body composition

RI, Rapid Inflator Cuff (Hokanson); PT, Personalized Tourniquet (Delfi PTS); HS, Handheld Sphygmanometer (Occlusion Cuff); LOP, Limb Occlusion Pressure; RPE, Rating of Perceived Exertion; MAP, Mean Arterial Pressure; DEXA, Dual-Energy X-ray Absorptiometry.

## 3 The oversight: BFR efficacy is already well-established

Despite limited evidence, considering the large variability between set and interface pressures that has been identified across devices, calls for improved stability between these variables seems reasonable, particularly as BFR is increasingly used in more and more clinical contexts (Hughes et al., 2024). Interface pressure is recognized as an important component of BFR application, yet consensus on how it should be measured remains lacking. Across the available studies, methods of assessing interface pressure vary substantially, and only Hughes et al. (2018) provided validation data to confirm the accuracy of their measurements. This lack of standardization complicates comparisons between studies and limits our ability to draw firm conclusions regarding the influence of interface pressure stability on training outcomes.

A substantial body of randomized-controlled trials and acute studies have implemented various BFR cuffs—ranging from autoregulated systems like the Delfi PTS to unregulated or older devices—across both clinical and athletic populations. Notably, many of these studies have used cuffs with unknown or inconsistent ability to maintain interface pressure, yet consistently report meaningful improvements in hypertrophy, strength, and function (Bjørnsen et al., 2019a; Bjørnsen et al., 2019b; Erickson et al., 2025; Jacobs et al., 2023; Ladlow et al., 2018; Lowery et al., 2014; Rolnick et al., 2024a). For example, Lowery et al. (2014) used subjective tightness with elastic straps and showed outcomes comparable to high-load training. Early et al. (2020) found similar benefits using B Strong cuffs, despite known pressure drift (Hughes et al., 2024). Kim et al. (2017) and Vechin et al. (2015) reported hypertrophy using devices not commercially designed for BFR, with no serious adverse events (Kim et al., 2017; Vechin et al., 2015). Direct comparisons using the Delfi PTS in autoregulated vs. unregulated configurations have shown minimal differences in repetition volumes, perceptual, or cardiovascular responses (Rolnick et al., 2024a; Rolnick et al., 2024c).

Taken together, these findings suggest that while interface pressure *may* be theoretically relevant, its precise regulation is not a prerequisite for effective BFR training. Adaptations appear robust across a spectrum of devices and protocols (Clarkson et al., 2024), underscoring the need for pragmatism rather than perfection in both clinical and performance settings (Fahs et al., 2012; Loenneke et al., 2012). While real-time pressure control may enhance consistency or safety tracking, the current evidence does not demonstrate a compelling need for this level of technological precision.

## 4 Real-world considerations

As BFR use continues to expand beyond laboratories and rehabilitation clinics, it is important to consider real-world factors like adherence and usability, and to critically assess how prioritizing features such as interface pressure may influence its broader application. While Hughes et al. (2018) demonstrated differences between devices in their ability to maintain set/interface pressure—and highlighted distinct effects on hemodynamic and perceptual responses—these differences may be of minimal concern in healthy populations, so long as the device/protocol elicits a meaningful BFR stimulus.

In many real-world settings (e.g., performance PT clinics, commercial, or home-based gyms), precise control of interface pressure may be neither feasible nor necessary, especially given the demonstrated efficacy of a wide range of BFR cuff pressures and protocols (Clarkson et al., 2024; Loenneke et al., 2025). Practitioners in athletic or performance settings often juggle multiple priorities and may benefit more from simple, reliable tools that facilitate consistent use—rather than marginal gains in pressure precision—particularly given that the primary goal of BFR is to accelerate fatigue and stimulate high-threshold motor unit recruitment (Loenneke et al., 2025).

That said, future research exploring interface pressure may help clarify its role in BFR’s physiological mechanisms, safety profile, and device-specific nuances. Recently, we proposed a BFR reporting guideline to improve transparency in describing device features, including set and interface pressure capabilities (Hughes et al., 2025). However, these guidelines aim to enhance methodological clarity—not to limit investigation to only devices with real-time pressure regulation. Emphasizing accessibility and relevance alongside scientific rigor is essential for BFR to continue evolving across both research and practical domains.

Importantly, there is currently no gold standard for assessing interface pressure, underscoring the need for validation studies to determine appropriate tools that accurately reflect the mechanical forces applied to the limb. Compounding this issue is the widespread use of mmHg to quantify cuff pressure—an indirect proxy—rather than directly measuring the mechanical force exerted on the limb.

Given this, it may be more appropriate to express interface pressure in units of force per area (e.g., N/cm^2^). Moreover, interface pressure is typically measured at a single location, without accounting for potential variation around the limb’s circumference. While devices like the Delfi PTS may regulate pressure effectively at the measurement site, there is limited evidence confirming that this regulation is consistent across the entire cuff-limb interface. Future research should examine whether localized readings accurately represent total limb compression forces and evaluate whether such variability impacts safety, comfort, or training efficacy.

## 5 A practical middle ground: tiered approach

Given the variability in device feature accessibility, population needs, and clinical contexts, a tiered model may offer a balanced framework for integrating interface pressure considerations into the BFR research literature and real-world application. In summary, practitioners should employ devices demonstrated to maintain consistent set/interface pressure in situations where precision is essential, or risk is elevated.

### 5.1 Tier 1 – precision-dependent applications

In high-risk or medically complex populations (e.g., post-operative cardiac rehabilitation)—non-elastic devices with autoregulatory capacity and real-time pressure feedback may offer added safety and dosing consistency across time. These contexts may also align with institutional liability concerns, where pressure traceability may be a priority.

During controlled laboratory investigations comparing the acute and longitudinal perceptual and psychophysiological effects of different devices and features, researchers should prioritize detailed reporting of cuff type, pressure methodology, and user guidance (Hughes et al., 2025). Importantly, recent calls for enhanced standardization in BFR reporting practices represent a welcome advancement and should be widely adopted across future studies.

### 5.2 Tier 2 – general population and performance settings

For healthy low-risk individuals, athletes, or general fitness applications, simpler tools—including semi-elastic multi-chambered systems or even strap-based devices—may suffice, provided that established effective BFR protocols are followed, and proper safety education is provided. These devices offer greater accessibility and have demonstrated efficacy across various applications despite variable interface pressure characteristics (Early et al., 2020; Hughes et al., 2024).

In short, device-specific characteristics should be acknowledged to improve standardization and deepen our understanding of how device features influence safety, efficacy, and usability. For example, recent work has demonstrated differential responses between autoregulated single-chambered systems and semi-elastic multi-chambered devices (Rolnick et al., 2025; Rolnick et al., 2024b) suggesting that physiological outcomes are mediated not solely by differences in pressure, but by the broader interface of device design, exercise prescription, and user effort.

## 6 Conclusion

Interface pressure is a valuable and emerging feature in the BFR literature, with important implications for standardization, safety, and dosing consistency—particularly in vulnerable populations and in studies aiming to distinguish the effects of device-specific features. However, using devices that maintain consistent set/interface pressure is not a universal requirement for effective or safe BFR implementation. While interface pressure stability is a useful feature—especially for standardization and dosing accuracy—it currently lacks sufficient empirical support to serve as a gatekeeping criterion. The robust body of evidence supporting the safety and efficacy of BFR across diverse devices and settings suggests that effective BFR practice is not contingent on a single device standard, but rather on a thoughtful consideration of each device’s strengths and limitations. By situating interface pressure within the broader ecosystem of variables—including cuff design, device features, user effort, and population-specific needs—BFR research and practice can continue to evolve in ways that prioritize safety, efficacy, accessibility, and innovation.
